# Gestational diabetes in women living with HIV in the UK and Ireland: insights from population‐based surveillance data

**DOI:** 10.1002/jia2.26078

**Published:** 2023-04-03

**Authors:** Laurette L. Bukasa, Mario Cortina‐Borja, Helen Peters, Graham P. Taylor, Claire Thorne

**Affiliations:** ^1^ Population, Policy and Practice Research & Teaching Department UCL Great Ormond Street Institute of Child Health London UK; ^2^ Section of Virology, Department of Infectious Disease Imperial College London London UK

**Keywords:** antiretroviral therapy, birth outcomes, gestational diabetes, HIV, pregnancy, women

## Abstract

**Introduction:**

The prevalence of gestational diabetes (GD) is increasing globally. While universal risk factors for GD are reasonably well understood, questions remain regarding risks for women living with HIV (WLWH). We aimed to describe GD prevalence, evaluate associated maternal risk factors and assess specific birth outcomes in WLWH in the UK and Ireland.

**Methods:**

We analysed all pregnancies (≥24 weeks’ gestation) in women diagnosed with HIV before delivery, reported to the UK‐based Integrated Screening Outcomes Surveillance Service between 2010 and 2020. Every report of GD was considered as a case. A multivariable logistic regression model, adjusted for women with more than one pregnancy fitted with generalized estimating equations (GEE) assessed the effect of independent risk factors.

**Results:**

There were 10,553 pregnancies in 7916 women, of which 460 (4.72%) pregnancies had reported GD. Overall, the median maternal age was 33 years (Q1:29–Q3:37), and 73% of pregnancies were in Black African women. WLWH with GD (WLWH‐GD) were older (61% vs. 41% aged ≥35 years, *p* < 0.001) and more likely to be on treatment at conception (74% vs. 64%, *p* < 0.001) than women without GD. WLWH‐GD were more likely to have a stillbirth (odds ratio [OR]: 5.38, 95% CI: 2.14–13.5), preterm delivery (OR: 2.54, 95% CI: 1.95–3.32) and fetal macrosomia (OR: 1.14, 95% CI: 1.04–1.24). Independent risk factors for GD included estimated year of delivery (GEE‐adjusted odds ratio [GEE‐aOR]: 1.14, 95% CI: 1.10–1.18), advanced maternal age (≥35 years) (GEE‐aOR: 2.87, 95% CI: 1.54–5.34), Asian (GEE‐aOR: 2.63, 95% CI: 1.40–4.63) and Black African (GEE‐aOR: 1.55, 95% CI: 1.13–2.12) ethnicity. Timing and type of antiretroviral therapy showed no evidence of a relationship with GD in multivariable analyses; however, women with a CD4 count ≤350 cells/μl were 27% less likely to have GD than women with CD4 counts >350 cells/μl (GEE‐aOR: 0.73, 95% CI: 0.50–0.96).

**Conclusions:**

GD prevalence increased over time among WLWH but was not significantly different from the general population. Maternal age, ethnicity and CD4 count were risk factors based on available data. Stillbirth and preterm delivery were more common in WLWH‐GD than other WLWH over the study period. Further studies are required to build upon these results.

## 1 INTRODUCTION

The risk of gestational diabetes (GD) is increasing proportionally to type 2 diabetes in the general population and now affects an estimated 16% of pregnancies globally [1]. Pregnancy is a physiological state of reduced insulin sensitivity [2] and GD is thought to result from complex mechanisms involving beta‐cell dysfunction influenced by genetic, epigenetic and environmental factors [3]. Pregnant women in the UK considered to be “at risk” of developing GD are selectively screened at 24–28 weeks’ gestation based on body mass index (BMI) >30 kg/m^2^, previous macrosomic baby (>4.5 kg), family history of diabetes and belonging to a minority ethnic group with a high prevalence of diabetes [4]. In 2015, clinical guidelines recommended a fasting plasma glucose ≥5.6 mmol/l or 2‐hour glucose on the oral glucose tolerance test ≥7.8 mmol/l for GD diagnosis, which differs from international criteria (≥5.1 and ≥8.5 mmol/l) and was lowered from ≥7.0 mmol/l for fasting plasma glucose in 2008 based on findings from the HAPO study [5]. While the universal risk factors for GD are reasonably well understood forming the basis of screening criteria, questions remain regarding the risk environment for women living with HIV (WLWH).

In the UK, there are approximately 800 pregnancies in WLWH each year, with 82% resulting in a delivery [6]. The vertical transmission rate has decreased by 90%, from 2.1% in 2000–2001 to 0.22% in 2017–2018 [7]. Guidelines for HIV management, including in pregnancy, have evolved over time alongside the development of new antiretroviral drugs and classes, influencing both the timing of treatment initiation and drugs used in pregnancy [8]. Since 2015 and the onset of the universal treatment era, women have increasingly been on treatment prior to conception [9]; for example, in the UK, 81% of pregnancies were conceived on antiretroviral therapy (ART) in 2018 [10]. Furthermore, there have been changes in the characteristics of pregnant WLWH, including increasing maternal age, a growing number with vertically acquired HIV and a decreasing proportion born in sub‐Saharan Africa [11, 12]. The implications of lifelong treatment for non‐pregnant populations may include increased risk of diabetes [13, 14] and cardiovascular disease [15, 16], but findings on the effect of treatment in pregnancy, particularly protease inhibitors (PIs), on GD risk have been inconsistent [17, 18, 19, 20]. Using population‐based data from the UK and Ireland from 2010 to 2020, our aim was to describe the prevalence of GD in WLWH, assess associated maternal risk factors and examine specific birth outcomes of pregnancies affected by GD.

## 2 METHODS

### 2.1 Integrated Screening Outcomes Surveillance Service

Integrated Screening Outcomes Surveillance Service (ISOSS) is commissioned by NHS England as part of the NHS Infectious Diseases in Pregnancy Screening Programme to conduct comprehensive population‐based active surveillance of all pregnancies to diagnosed WLWH in the UK and Ireland since 1989 (formerly the National Study of HIV in Pregnancy and Childhood) [10]. Data from Ireland are included until 2018, and Scotland, Wales and Northern Ireland included until 2019 [6]. Universal screening for HIV in pregnancy has been a standard of antenatal care since 2001 [21] and maternity reports of all pregnancies in WLWH are submitted by antenatal units at notification (antenatal booking) and at outcome (delivery); they include information on socio‐demographics, ART (type and timing), obstetric management and mode of delivery (emergency caesarean section [CS], elective CS and vaginal). Pregnancy complications were defined as any pre‐existing (i.e. diabetes and hypertension) or incident (e.g. GD, pre‐eclampsia) conditions. Reports are submitted by a named responder in each maternity unit via a secure online portal [10]. All data are collected as part of routine antenatal care and selected data are shared with ISOSS without patient consent under Regulation 3 of The Health Service (Control of Patient Information) Regulations 2002 [22]. This research involved the secondary use of ISOSS data and received approvals from the West Midlands—Solihull Research Ethics Committee (21/WM/0040) and the Antenatal and Newborn Screening Programme Research Advisory Committee (ANNB_IDPS_0037).

### 2.2 Analysis dataset

Analyses were conducted on a pseudonymized dataset of all pregnancies in women with HIV diagnosed before delivery (i.e. before and during pregnancy) with a reported outcome ≥24 weeks’ gestation and estimated date of delivery (EDD) between 1 January 2010 and 30 June 2020 reported by September 2020. Women with a HIV‐2 diagnosis, and women with a multiple pregnancy were excluded.

**Figure 1:**
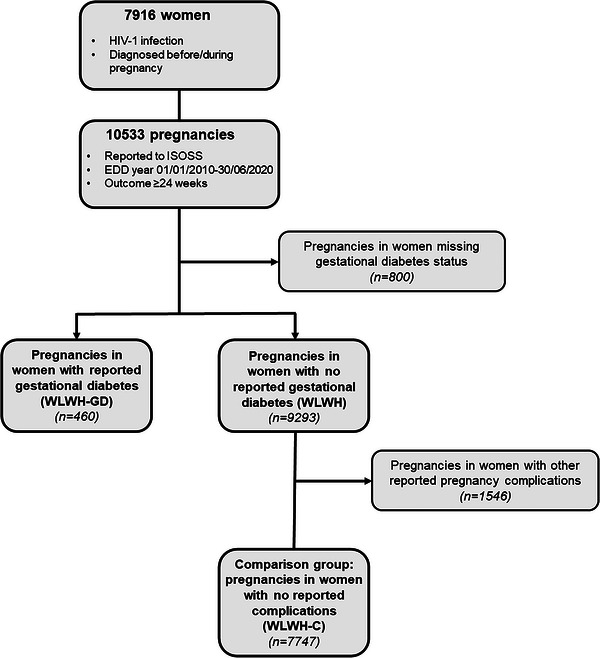
Overview of study population.

Descriptive analyses used a dataset, including pregnant women with reported GD and pregnancies without a report of GD. Analyses of adverse birth outcomes and risk factors for GD used a dataset, including women with GD and a comparison population of women without GD or any other pregnancy complication reported, Figure 1.

### 2.3 Definitions and classifications

All reports of GD were classified as a case (WLWH‐GD). We considered ART use (Yes/No) and any use of the main third agent classes (Non‐Nucleoside Reverse Transcriptase Inhibitor (NNRTI), Integrase Strand Transfer Inhibitor (INSTI) and PI) in pregnancy (i.e. if a woman had a third agent drug switch in pregnancy, she would be included in all the third agent classes used). HIV‐1 RNA viral load (VL) results were recorded at antenatal booking and delivery (from 36 weeks gestation to 7 days postpartum) where available. ISOSS requests a minimum of one CD4 count but up to two VL test results can be reported. We defined the first CD4 count as the earliest reported measurement between 8 weeks before the last menstrual period (LMP) and 1 week after delivery, categorized as ≤350 and >350 cells/mm^3^. LMP was estimated as 280 days prior to EDD. Coinfections were defined as reported hepatitis B, hepatitis C or syphilis, with other infections provided ad hoc. HIV clinical status relates to reported symptoms in pregnancy, including AIDS events. Parity since diagnosis was estimated for each woman based on the number of pregnancies ever recorded in ISOSS as a proxy.

Macrosomia was defined as birthweight ≥4.00 kg and preterm delivery as occurring at <37 gestational weeks. Stillbirth was defined as an intrauterine death occurring at ≥24 gestational weeks. Adverse birth outcomes were described by year group (2010–2014 vs. 2015–2020) to explore the effect of both the universal HIV treatment era (from 2015) and the lowered fasting plasma glucose diagnostic threshold for GD.

### 2.4 Risk factor analyses

The EDD year, maternal socio‐demographics (age at delivery, ethnicity, region of origin, parity since diagnosis) and maternal HIV‐related characteristics (risk factor for HIV acquisition, timing of diagnosis, coinfections, clinical status, treatment at conception, type of treatment, VL and CD4 count in pregnancy) were included in univariable analyses.

The multivariable independent maternal effects model included covariates based on univariable analyses. Multicollinearity within the model was assessed using the variance inflation factor [23]. All covariates were included in model selection using penalized regression methods with ART at conception and PI use in pregnancy remaining in all estimation models *a priori*. Covariates were included based on the most parsimonious model accounting for existing knowledge on possible risk factors.

To account for women with more than one pregnancy in the study, the multivariable independent maternal effects regression model was fitted using generalized estimating equations (GEE). The quasi‐likelihood information criterion was used to assess the goodness of fit [24] in a complete‐case analysis of independent effects. Sensitivity analyses were conducted to evaluate time‐related effects on risk factors for GD and the effect of women with >1 pregnancy affected by GD.

All univariable analyses were conducted in STATA/SE 15.1 (StataCorp LLC, College Station, Texas, USA), with multivariable analyses conducted in R 4.0.4 (R Core Team (2020)).

## 3 RESULTS

Of the 10,553 pregnancies in 7916 women reported by September 2020, 800 (7.6%) pregnancies were missing GD status and not included in analyses (Figure 1). Pregnancies in WLWH who were missing GD status were more likely to have been pregnant before 2015, to have acquired HIV by injecting drug use (*p* < 0.001), been diagnosed during pregnancy (*p* = 0.018) and received PI‐based treatment (*p* < 0.001); maternal age and ethnicity did not differ between pregnancies with and without GD status (Table S1). In the remaining 9753 pregnancies, 460 (4.72%, 95% CI: 4.30%, 5.16%) pregnancies were in women with reported GD and 9293 in women without reported GD, of which 7747 pregnancies were included in a comparison group with no reported pregnancy complications (WLWH‐C), Figure 1.

**Figure 2:**
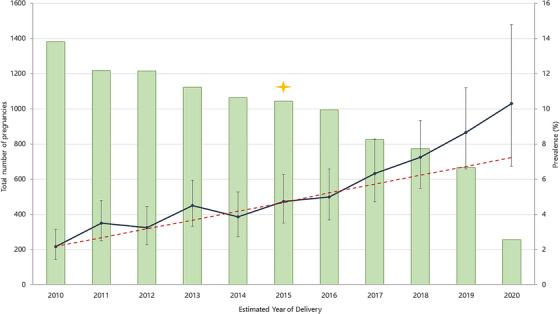
Prevalence of gestational diabetes in pregnancies in women living with HIV between 2010 and June 2020. Green bars correspond to the total number of pregnancies; blue/grey solid line corresponds to the prevalence of GD with error bars representing 95% confidence intervals for the proportion; red dashed line is the predicted prevalence of GD based on the average percentage increase observed in 2010–2014. Yellow star indicates the year when the GD fasting glucose threshold was lowered and the universal HIV treatment era began.

Twenty‐eight women had >1 GD pregnancy, contributing 59 (12.8%) of the total 460 cases; of all GD pregnancies in women from Asian ethnic backgrounds, 20.8% (5/24) were in women with >1 GD pregnancy, compared to 14.3% (50/348) of women from Black ethnic backgrounds (*p <* 0.001). Overall, GD prevalence increased from 3.39% (95% CI: 2.92–3.91) in 2010–2014 to 6.39% (95% CI: 5.68–7.17) in 2015–2020 with half (49.8%, 229/460) of all GD cases reported since 2016 (Figure 2).

Median maternal age was 33 years (IQR: 8, Q1:29–Q3:37), with 71.8% of pregnancies in Black African women and 64% of pregnancies conceived on treatment (Table 1). For WLWH with timing of ART initiation available, 96.4% (9901/10,266) had received treatment before the third trimester. PIs were the most common third agent (6188/10,553, 58.6%). A similar number of pregnancies were reported during the study period within the COVID‐19 pandemic (March–June 2020, *N* = 185) compared to the previous four months (November 2019–February 2020, *N* = 186); GD prevalence across both periods among pregnancies with a GD status was comparable (13.1% [23/175] vs. 8.67% [15/173], *p* = 0.185).

**Table 1:** Maternal characteristics of pregnancies delivered in 2010–2020 with gestational diabetes status.

Risk factor	*n*	Frequency	(%)
Age (years)	9752		
<25		736	(7.55)
25—34		7938	(50.6)
≥35		4078	(41.8)
Maternal ethnic background	9730		
White		1824	(18.8)
Black African		7003	(72.0)
Black Caribbean		317	(3.26)
Asian		284	(2.92)
Other		302	(3.10)
Maternal origin	9645		
UK/Ireland		1580	(16.4)
Africa		6833	(70.8)
Elsewhere		1232	(12.8)
Woman's risk factor for acquisition	9056		
Heterosexual		8628	(95.3)
IDU		111	(1.23)
Vertical		135	(1.49)
Other^a^		182	(2.01)
Timing of diagnosis	9753		
Before		8324	(85.4)
During		1429	(14.7)
On treatment at conception	9686		
Yes		6229	(64.3)
No		3457	(35.7)
PI in pregnancy	9695		
Yes		5677	(58.6)
No		4018	(41.4)
NNRTI in pregnancy	9695		
Yes		3315	(34.2)
No		6380	(65.8)
INSTI in pregnancy	9695		
Yes		1547	(16.0)
No		8148	(84.0)
First VL in pregnancy	9648		
<50		5295	(54.9)
51–9999		2876	(29.8)
≥10,000		1477	(15.3)
CD4 count in pregnancy (cells/μl)	8998		
<350		2380	(26.5)
≥350		6618	(73.6)

Abbreviations: INSTI, integrase strand transcriptase inhibitor; NNRTI, non‐nucleoside reverse transcriptase inhibitor; PI, protease inhibitor; VL, viral load.

^a^ Other risk factors for acquisition included blood transfusion and contact with infected blood.

Overall, 51.3% (235/458) of pregnancies in WLWH‐GD were exposed to PIs, with 82.6% exposed from conception (i.e. no drug switches). An increasing proportion of WLWH‐GD were on treatment at conception over time (61.4% [62/101] in 2010–2012 vs. 82% [187/228] in 2016–2020). PI‐based regimens were used less frequently over time, decreasing from 70.0% (70/100) in 2010–2012 to 42.7% (97/227) in 2016–2020. Over time, an increasing proportion of WLWH‐GD were aged ≥35 years (49.5% [50/101] in 2010–2012 vs. 61.8% [141/228] in 2016–2020, *p* < 0.001) and had an undetectable first VL in pregnancy (48.5% [49/101] in 2010–2012 vs. 66.8% [153/229] in 2016–2020, *p* = 0.018).

There were no statistically significant differences between pregnancies in WLWH and WLWH‐GD regarding exposure to any ART in pregnancy (*p* = 0.774), concurrent infections (*p* = 0.349) and HIV clinical status (*p* = 0.390), but WLWH‐GD were more likely to have initiated ART before the third trimester (*p* < 0.001), less likely to have received ≥3 drug classes (*p* = 0.573) and have a lower first VL in pregnancy (*p* = 0.016) than other WLWH (Table S2).

Adverse birth outcomes were described for 8207 pregnancies: 460 pregnancies with GD and 7747 pregnancies in WLWH‐C (Table 2). Pregnancies in WLWH‐GD were more likely to end in stillbirth (Odds ratio (OR): 5.38, 95% CI: 2.14–13.5) and preterm delivery (OR: 2.54, 95% CI: 1.95–332) than WLWH‐C. Emergency CS (OR: 1.54, 95% CI: 1.22–1.94) compared to elective CS, and fetal macrosomia (OR: 1.14, 95% CI: 1.04–1.24) were also more common among WLWH‐GD than WLWH‐C. When stratified by time period, all statistically significant associations between GD and adverse birth outcomes remained aside from fetal macrosomia (Table 2).

**Table 2:** Selected outcomes of pregnancies affected by gestational diabetes compared to a control group of pregnancies in women without GD or other pregnancy complications.

						2010–2014 (*N* = 4712)					2015–2020 (*N* = 3495)				
	Pregnancies in WLWH‐GD *N =* 460		Pregnancies in WLWH‐C *N =* 7747			Pregnancies in WLWH‐GD *N =* 185		Pregnancies in WLWH‐C *N =* 4527			Pregnancies in WLWH‐GD *N =* 275		Pregnancies in WLWH‐C *N =* 3220		
*Outcome*	*n*	*(%)*	*n*	*(%)*	*p‐value* ^a^	*n*	*(%)*	*n*	*(%)*	*p‐value* ^a^	*n*	*(%)*	*n*	*(%)*	*p‐value* ^a^
**Pregnancy outcome** ^b^															
Livebirth	454	(98.7)	7728	(99.8)	0.038	182	(98.4)	4513	(99.7)	0.004	272	(98.9)	3215	(99.8)	0.002
Stillbirth	6	(1.30)	19	(0.25)		3	(1.62)	14	(0.31)		3	(1.09)	5	(0.16)	
**Mode of delivery**															
Emergency CS	154	(33.5)	1583	(20.5)	<0.001	70	(37.8)	950	(21.0)	<0.001	84	(30.6)	603	(19.7)	<0.001
Elective CS	153	(33.3)	2422	(31.3)		64	(34.6)	1467	(32.5)		89	(32.4)	955	(29.7)	
Vaginal	153	(33.3)	3733	(48.2)		51	(27.6)	2101	(46.5)		102	(37.1)	1632	(50.8)	
**Gestation (weeks)**															
37+	388	(84.4)	7220	(93.2)	<0.001	148	(80.0)	4216	(93.1)	<0.001	240	(87.3)	3004	(93.3)	0.001
33–36	61	(13.3)	423	(5.46)		31	(16.8)	249	(5.50)		30	(10.9)	174	(5.40)	
<33	11	(2.39)	104	(1.34)		6	(3.24)	62	(1.37)		5	(1.82)	42	(1.30)	
**Fetal macrosomia**															
Yes	37	(8.20)	388	(5.10)	<0.001	19	(10.6)	206	(4.64)	<0.001	18	(6.62)	182	(5.74)	0.292
No	414	(91.8)	7222	(94.9)		160	(89.4)	4231	(95.4)		254	(93.4)	2991	(94.3)	

^a^ Chi‐squared test.

^b^ Total does not equal 8702 for pregnancies in WLWH because of a late TOP at 25 weeks that was neither a livebirth nor stillbirth.

A GEE model with an exchangeable correlation structure was superior to other correlation structures and was used in the independent effects model based on a complete case analysis of pregnancies (6880/8207) in WLWH‐GD and WLWH‐C. After adjusting for covariates, the odds of GD increased by 14% each year over the study period, Table 3. Pregnancies in women aged ≥35 years (GEE‐adjusted odds ratio (aOR): 2.87, 95% CI: 1.54–5.34) and from Asian (GEE‐aOR: 2.60, 95% CI: 1.46–4.63), Black Caribbean (GEE‐aOR: 2.63, 95% CI: 1.40–4.93) and Black African (GEE‐aOR: 1.55, 95% CI: 1.13–2.12) backgrounds were at greater risk of developing GD compared to women aged <25 years and women from White backgrounds, respectively. Low CD4 cell count (<350) was associated with reduced odds of developing GD (GEE‐aOR: 0.73, 95% CI: 0.56–0.96). No relationships were observed for treatment at conception or PI use. The independent maternal effects model with GEE was fitted for 5576 clusters (max size: 5) with some evidence of population‐level within‐cluster correlation for women with multiple pregnancies (α = 0.307, *p* = 0.06).

**Table 3:** Risk factor analyses for gestational diabetes in univariable (*N* = 8207) and multivariable GEE (*N* = 6880) models.

	WLWH‐GD *n* = 460		WLWH‐C *n* = 7747					
Risk factor	*n*	(%)	*n*	(%)	OR	(95% CI)	GEE‐aOR	(95% CI)
Year of estimated date of delivery^a^					1.16	(1.12–1.20)	1.14	(1.10–1.18)
2010–2012	101	(22.0)	2934	(37.9)	–	–	–	–
2013–2015	130	(28.3)	2324	(30.0)	–	–	–	–
2016–2020^b^	229	(49.8)	2489	(32.1)	–	–	–	–
Maternal age at delivery (years)								
<25	13	(2.83)	611	(7.89)	1		1	
25–34	167	(36.3)	4091	(52.8)	1.92	(1.08–3.40)	1.51	(0.82–2.80)
≥35	280	(60.9)	3045	(39.3)	4.32	(2.46–7.59)	2.87	(1.54–5.34)
Maternal ethnicity								
White	57	(12.5)	1491	(19.3)	1		1	
Black African	348	(76.0)	5521	(71.4)	1.65	(1.24–2.19)	1.55	(1.13–2.12)
Black Caribbean	16	(3.49)	250	(3.23)	1.67	(0.94–2.96)	1.72	(0.94–3.14)
Asian	24	(5.24)	231	(2.99)	2.72	(1.65–4.47)	2.63	(1.40–4.63)
Other	13	(2.84)	238	(3.08)	1.43	(0.77–2.65)	1.09	(0.50–2.38)
Parity since diagnosis								
1	169	(36.7)	2359	(30.5)	1		1	
2	146	(31.7)	2633	(34.0)	0.77	0.62–0.97	0.81	(0.62–1.06)
3	92	(20.0)	1731	(22.3)	0.74	0.57–0.96	0.76	(0.56–4.64)
≥4	53	(11.5)	1024	(13.2)	0.72	0.53–0.99	0.75	(0.51–1.11)
Maternal risk factor for HIV acquisition								
Heterosexual	415	(96.7)	6868	(95.4)	1		1	
Injecting drug use	4	(0.93)	79	(1.10)	0.84	0.31–2.30	1.61	(0.56–4.64)
Vertical	0		150	(2.08)	1		1.69	(0.77–3.71)
Other	10	(2.33)	101	(1.40)	1.64	0.85–3.16	1	
Maternal CD4 count (cells/μl)								
≥350	336	(81.0)	5621	(73.5)	1		1	
<350	79	(19.0)	1889	(26.5)	0.65	(0.51–0.84)	0.73	(0.56–0.96)
On treatment at conception								
No	121	(26.4)	2822	(36.7)	1		1	
Yes	338	(73.6)	4874	(63.3)	1.62	(1.31–2.00)	1.05	(0.82–1.36)
PI use in pregnancy								
No	223	(48.7)	3176	(41.2)	1		1	
Yes	235	(51.3)	4532	(58.8)	0.74	(0.61–0.89)	0.96	(0.76–1.20)

^a^ Year included as a continuous variable in logistic regression model.

^b^ Data reported by September 2020 for deliveries occurring 1 January 2010 and 30 June 2020.

When women with more than one GD pregnancy were excluded (*n* = 8148), EDD year, age, ethnicity and CD4 count remained significant risk factors; additionally, multiparous WLWH since HIV diagnosis were less likely to have a pregnancy affected by GD (Table S3). Advanced maternal age (≥35 years) (*p* < 0.001) and Asian ethnicity (*p* < 0.001) remained significant risk factors for GD in sensitivity analyses across both 2010–2014 (*n* = 4712) and 2015–2020 (*n* = 3495) time periods, while the association with CD4 count was only present in 2010–2014 (*p* = 0.107), Table S3. Pregnancies in WLWH from Black African and Black Caribbean ethnic backgrounds were at greater risk of GD compared to women from White ethnic backgrounds in the 2010–2014 period alone.

## 4 DISCUSSION

UK and Ireland national surveillance data were used to characterize and identify risk factors for pregnant WLWH who were affected by GD delivering between 2010 and 2020. There were higher rates of treatment at conception, undetectable VL and CD4 cell counts ≥350 in WLWH‐GD pregnancies compared to WLWH without GD or other pregnancy complications. The odds of GD increased by 14% each year even after adjusting for the ageing and increasingly multiparous population. Pregnancies with GD were more likely to be in women from minority ethnic groups than White women, and in women with CD4 counts ≥350 cells/μl.

The overall prevalence of GD in our study population (4.72%) was comparable with estimates from the general population in the UK [25, 26]. The increasing GD prevalence over time (reaching 8.7% by 2019) may be attributed to concurrent changes in HIV‐related and universal factors, including HIV/ART effects on insulin resistance [27, 28], the reduction in the fasting plasma glucose threshold for GD diagnosis [4], and increasing obesity observed in both the general population [29] and WLWH [30]. Our prevalence estimates are comparable with those reported for WLWH in other high‐income settings (2.6–11.4%) [17, 31, 32, 33]; however, comparisons must be made with caution given differences in diagnostic criteria. As increases in GD prevalence are observed in the general obstetric population both in high‐income [34] and low‐income settings [35], challenges remain in determining whether an excess risk exists for WLWH.

While only capturing data on pregnancies in the first 4 months of the COVID‐19 pandemic, GD prevalence was comparable with within‐study estimates from November 2019 to February 2020 and general population estimates based on hospital admissions data (8.3%) [36], despite possible underestimation due to deviation from standard screening guidelines [37].

Few studies of WLWH have explored temporal trends in GD prevalence, although findings from earlier periods (1990–2006) reported an upward trend with 2–3% increases over time, with increased coverage with ART considered a contributing factor [38, 39]. Here, the temporal increase in GD prevalence persisted despite adjustment for timing and type of ART alongside universal risk factors, such as ethnicity. Use of ART in pregnant WLWH has changed significantly over the study period from treatment by indication (CD4 < 350) [40] to lifelong treatment from diagnosis [41]; however, sensitivity analyses showed that neither ART use at conception nor use of PIs in pregnancy were associated with GD in these two treatment eras when considered individually. Recent studies have also reported similar findings [42, 43], although PI‐based regimens were implicated in earlier studies [17, 18] with a meta‐analysis suggesting that this association may be limited to studies where first‐generation PI and the strictest GD diagnostic criteria were used [20].

The use of newer drugs in pregnancy, such as some integrase inhibitors, could become increasingly relevant for GD risk in the future although insufficient sample sizes precluded analyses here. Evidence is currently accumulating on the effects of ART on fat mass accrual both in and outside of pregnancy [30]. A comparative analysis of randomized clinical trials has shown that weight gain is greater in more recent trials with the use of newer ART regimens [44], which is consistent with post‐marketing observational studies of integrase inhibitors [45]. Preliminary studies of WLWH on integrase inhibitors during pregnancy show no cause for concern regarding gestational weight gain [42, 46]; however, a risk prediction model based on data from South Africa suggests that treatment emergent obesity with dolutegravir‐based regimens (with tenofovir alafenamide or disoproxil and emtricitabine) for obese women (BMI ≥30 kg/m^2^) versus those with normal BMI could increase the risk of GD four‐fold (RR: 4.31, 95% CI: 3.18–5.85) [47].

Women with CD4 counts <350 were less likely to develop GD than other women in our study. Restoration of the immune system through ART, approximated by higher CD4 counts, could increase the risk of some pregnancy complications [48]. The “return to health” effect is associated with increases in CD4 counts and weight gain [44], while lower CD4 counts are associated with lower weight and could potentially reflect poorer ART adherence [49, 50]. An alternative pathway could involve reduced albumin levels (associated with lower CD4 counts in the non‐pregnant population [51]), given the link between high albumin levels and insulin resistance [52].

Advanced maternal age has been associated with GD both in the general population [53, 54] and among WLWH [17, 55, 56]. Women ≥35 years were most likely to develop GD in our population, which could be a proxy for age‐related weight gain [57]. A recent study in Botswana reported similar findings with GD risk increasing linearly with each year of age (aOR: 1.10, 1.04–1.17) [42]. Maternal age is also related to gravidity, which was accounted for in the modelling strategy; however, evidence from the general population indicates that the incidence of GD is three‐ to six‐fold higher among women over 40 years of age [58], and a significant proportion of women who develop GD in their first pregnancy are at greater risk of developing GD in subsequent pregnancies [59, 60]. Approximately 6.5% of women with GD had more than one GD pregnancy, which is substantially lower than recurrent rates of 30–60% reported in the general pregnant population [59, 61, 62]; this was likely due to the restricted study period and lack of data on previous pregnancies prior to HIV diagnosis or arrival in the UK that may have been affected by GD. WLWH from Asian backgrounds (representing only 3% of our study population) were at the greatest risk of GD, consistent with global pooled prevalence estimates [63]. Similarly, women from Black African backgrounds were approximately 1.5 times more likely to have GD than White women, consistent with women belonging to this ethnic group being identified as “at risk” of GD in the general population [4].

Pregnancies in WLWH‐GD were more likely to have adverse birth outcomes compared to women without reported pregnancy complications (WLWH‐C). While prevalence of adverse birth outcomes among WLWH‐GD from 2010–2014 to 2015–2020 declined, stillbirth, preterm delivery and CS remained more common among WLWH‐GD than WLWH‐C. These findings are comparable to results from studies in the general population that indicate that women who develop GD in pregnancy are more likely to have a preterm delivery [64] and have a CS [65], partly due to fetal macrosomia [66]. The CS rate in our study was similar to the general population in other high‐income countries, but the preterm delivery and stillbirth rate were greater [67]. Stillbirths among WLWH‐GD (1.30%) were five times greater than WLWH‐C (0.25%) and the general population (0.3%) [68]. Evidence on stillbirth risk for women affected by GD in the general population is conflicting [69, 70]; however, among WLWH, strong relationships between GD and stillbirth have been reported in the UK with a 2.8‐fold increased incident rate ratio of stillbirth among women with GD [71]. A South African study of pregnant women with diabetes also reported elevated perinatal mortality in WLWH compared with other women (9.4% vs. 1.8%, *p* < 0.001) [72]. Collectively, these studies indicate that WLWH may require specific management.

While the use of population‐level surveillance data on pregnant WLWH was a study strength, it was also a source of limitations as several important variables were not collected. This includes BMI, a GD risk factor in the general population [73, 74] and among WLWH [42] that could not be accounted for in our analyses. Approximately 41% of WLWH in the UK are overweight [75], with an increasing trend in general obesity reported nationally [29], highlighting complex interactions with GD that could not be elucidated in our study. Similarly, any GD diagnoses in pregnancies outside of the study period, prior to HIV diagnosis and/or arrival in the UK were not available and could, therefore, not be included in adjusted analyses. In the absence of data on historical GD diagnoses, BMI and known risk factors for GD, such as family history of diabetes, we were unable to assess whether these factors influenced GD risk and any interaction with HIV‐related factors, such as ART use.

Our analyses of adverse birth outcomes and GD risk factors compared WLWH‐GD with a selected sub‐group of pregnancies without complications based on available data. A more appropriate control group would have been women not meeting the threshold for a GD diagnosis and without any known GD risk factors (for which we lacked data), as women “at risk” of GD based on screening criteria but who are not screened may have a greater risk of adverse birth outcomes than those screened [76]. Data were not available on the management of GD which could have impacted the relative risk of adverse birth outcomes [64].

## 5 CONCLUSIONS

WLWH do not appear to be disproportionately affected by GD compared to women in the general population. Our findings highlight that there are shared risk factors with the general population and, in the absence of data on other risk factors, maternal age, ethnicity and CD4 count are associated with GD. Timing and type of ART did not affect GD risk, but the lack of data on key risk factors, such as BMI, particularly in the context of newer drugs, warrants further investigation. Optimizing pregnancy and birth outcomes for WLWH is increasingly important in this low‐risk era for vertical transmission, as the risk of stillbirth and preterm delivery are substantially greater in WLWH‐GD despite updated clinical GD screening guidelines. Effective management of GD can reduce the risk of adverse outcomes [76] and thus further research on how GD is managed in this population is required to inform strategies for intervention, alongside future research to understand better the complex relationships between HIV infection and management, GD, pregnancy complications and birth outcomes.

## AUTHOR’S CONTRIBUTIONS

LLB and CT conceived this study. LLB wrote the original draft. LLB, GPT and MCB planned the statistical analyses. LLB performed the statistical analyses. HP and LLB were responsible for data acquisition, processing and verification. All authors contributed to interpreting the results and reviewing the article.

## FUNDING

This work is (partly) funded by the National Institute for Health and Care Research (NIHR) Great Ormond Street Hospital Biomedical Research Centre.

## COMPETING INTERESTS

The authors declare no competing interests.

## DISCLAIMER

The views expressed are those of the authors and not necessarily those of the NHS, the NIHR, the Department of Health or Public Health England.

## Supporting information




**Table S1**: Characteristics of pregnancies missing gestational diabetes (GD) status
**Table S2**: Characteristics of pregnancies in women with gestational diabetes and women without gestational diabetes
**Table S3**: Sensitivity risk factor analyses using independent maternal effects model restricted by women with only one reported GD event during study period and by year group

## Data Availability

The data that support the findings of this study are available from the NHS Infectious Diseases in Pregnancy Screening Programme via the Antenatal and Newborn Screening Research Advisory Committee. Restrictions apply to the availability of these data, which were used under license for this study. Data are available from the https://www.ucl.ac.uk/integrated‐screening‐outcomes‐surveillance/ with the permission of the Antenatal and Newborn Screening Research Advisory Committee (https://www.gov.uk/government/publications/annb‐screening‐submitting‐research‐proposals/annb‐screening‐research‐advisory‐committee).
