# Supplementary material for: Gestational diabetes in women living with HIV in the UK and Ireland: insights from population‐based surveillance data
Source: J Int AIDS Soc. 2023 Apr 3;26(4):e26078. doi: 10.1002/jia2.26078 (PMC10071091; doi:10.1002/jia2.26078)
Supplement: Supplementary file 1 — Table S1: Characteristics of pregnancies missing gestational diabetes (GD) status Table S2: Characteristics of pregnancies in women with gestational diabetes and women without gestational diabetes Table S3: Sensitivity risk factor analyses using independent maternal effects model restricted by women with only one reported GD event during study period and by year group [file JIA2-26-e26078-s001.docx]

**Supporting Information**

Supporting information - Table 1: Characteristics of pregnancies missing gestational diabetes (GD) status

|  | Missing GD status  (N=800) | | GD status available (N=9753) | | p-value |
| --- | --- | --- | --- | --- | --- |
| Estimated year of delivery |  |  |  |  |  |
| 2010-2012 | 369 | (9.68) | 3443 | (90.3) | <0.001 |
| 2013-2015 | 247 | (7.65) | 2980 | (92.4) |  |
| 2016-2020 | 184 | (5.24) | 3330 | (24.8) |  |
| Maternal age |  |  |  |  |  |
| <25 | 69 | (8.57) | 736 | (91.4) | 0.533 |
| 25-34 | 398 | (7.46) | 4938 | (92.5) |  |
| ≥35 | 332 | (7.53) | 4078 | (92.5) |  |
| Maternal ethnicity |  |  |  |  |  |
| White | 168 | (8.43) | 1824 | (91.5) | 0.600 |
| Black African | 558 | (7.38) | 7003 | (92.6) |  |
| Black Caribbean | 25 | (7.31) | 317 | (92.7) |  |
| Asian | 21 | (6.89) | 284 | (93.1) |  |
| Other | 25 | (7.65) | 302 | (92.4) |  |
| Risk factor for acquisition |  |  |  |  |  |
| Heterosexual | 670 | (7.21) | 8626 | (92.8) | <0.001 |
| Injecting drug use | 24 | (17.8) | 111 | (82.2) |  |
| Other | 13 | (8.78) | 135 | (91.2) |  |
| Vertical | 20 | (9.90) | 182 | (90.1) |  |
| Not known | 73 | (9.48) | 697 | (90.5) |  |
| Timing of diagnosis |  |  |  |  |  |
| Before | 658 | (7.33) | 8324 | (92.7) | 0.018 |
| During | 142 | (9.04) | 1429 | (91.0) |  |
| On treatment at conception |  |  |  |  |  |
| Yes | 462 | (6.90) | 6229 | (93.1) | 0.006 |
| No | 316 | (8.38) | 3457 | (91.6) |  |
| First viral load in pregnancy |  |  |  |  |  |
| <50 | 393 | (6.91) | 5296 | (93.1) | 0.207 |
| 51-9999 | 224 | (7.23) | 2876 | (92.8) |  |
| ≥10,0000 | 132 | (8.20) | 1477 | (91.8) |  |
| CD4+ T-cell in pregnancy |  |  |  |  |  |
| <350 | 203 | (7.86) | 2380 | (92.1) | 0.098 |
| ≥350 | 489 | (6.88) | 6618 | (93.1) |  |
| PI in pregnancy |  |  |  |  |  |
| Yes | 511 | (8.26) | 5677 | (91.7) | <0.001 |
| No | 275 | (6.41) | 4018 | (93.6) |  |
| NNRTI in pregnancy |  |  |  |  |  |
| Yes | 228 | (6.44) | 3315 | (93.6) | 0.003 |
| No | 558 | (8.04) | 6380 | (92.0) |  |

*p-value from chi-squared test

PI: protease inhibitor; NNRTI: non-nucleoside reverse transcriptase inhibitor

Supporting information - Table 2: Characteristics of pregnancies in women with gestational diabetes and women without gestational diabetes

|  | **Gestational Diabetes Mellitus** | | | |  |
| --- | --- | --- | --- | --- | --- |
|  | **Yes (%), N=460** | | **No (%), N=9293** | | **p-value*** |
| Maternal region of origin |  |  |  |  |  |
| UK/Ireland | 52 | (3.30) | 1528 | (96.7) | 0.012 |
| Africa | 344 | (5.03) | 6489 | (95.0) |  |
| Elsewhere | 56 | (4.55) | 1176 | (95.5) |  |
| Timing of diagnosis |  |  |  |  |  |
| Before | 405 | (4.87) | 7919 | (95.1) | <0.001 |
| During | 55 | (3.85) | 1374 | (96.2) |  |
| Exposure to ART in pregnancy |  |  |  |  |  |
| Yes | 458 | (4.72) | 9238 | (95.28) | 0.774 |
| No | 1 | (6.25) | 15 | (93.75) |  |
| Timing of ART initiation |  |  |  |  |  |
| Before pregnancy | 338 | (74.8) | 5891 | (65.0) | <0.001 |
| 1^st^ Trimester | 15 | (3.32) | 360 | (3.97) |  |
| 2^nd^ Trimester | 93 | (20.6) | 2491 | (27.5) |  |
| 3^rd^ Trimester | 6 | (1.33) | 324 | (3.57) |  |
| NNRTI use in pregnancy |  |  |  |  |  |
| Yes | 175 | (5.30) | 3140 | (94.7) | 0.063 |
| No | 283 | (4.44) | 6097 | (95.6) |  |
| INSTI use in pregnancy |  |  |  |  |  |
| Yes | 76 | (4.90) | 1471 | (95.1) | 0.703 |
| No | 382 | (4.69) | 7766 | (95.3) |  |
| Concurrent Infections |  |  |  |  |  |
| Yes | 41 | (9.90) | 981 | (87.95) | 0.189 |
| No | 373 | (90.1) | 7161 | (12.05) |  |
| HIV Clinical Status |  |  |  |  |  |
| Symptomatic | 3 | (99.3) | 105 | (1.21) | 0.325 |
| Asymptomatic | 434 | (0.69) | 8582 | (98.8) |  |
| First VL in pregnancy |  |  |  |  |  |
| <50 | 279 | (60.8) | 5016 | (54.6) | 0.016 |
| 51-9,999 | 127 | (27.7) | 2749 | (29.9) |  |
| ≥10,000 | 53 | (11.5) | 1424 | (15.5) |  |
| No. of drug classes used in pregnancy |  |  |  |  |  |
| 0 | 3 | (0.65) | 98 | (1.05) | 0.573 |
| 1 | 15 | (3.26) | 226 | (2.43) |  |
| 2 | 402 | (87.4) | 7985 | (85.9) |  |
| ≥3 | 40 | (8.69) | 984 | (10.6) |  |

*p-value from chi-squared test

Supporting information - Table 3: Sensitivity risk factor analyses using independent maternal effects model restricted by women with only one reported GD event during study period and by year group

|  | Women with only 1 GD pregnancy (N=6829) | | 2010-2014 (N=4066) | | 2015-2020 (N=2814) | |
| --- | --- | --- | --- | --- | --- | --- |
|  | **aOR** | **(95% CI)** | **aOR** | **(95% CI)** | **aOR** | **(95% CI)** |
| **EDD year** | 1.14 | (1.09-1.18) | 1.15 | (1.02-1.28) | 1.16 | (1.06-1.27) |
| **Age Group (years)** |  |  |  |  |  |  |
| <25 | 1 |  | 1 |  | 1 |  |
| 25-34 | 1.56 | (0.78-3.11) | 1.73 | (0.62-4.86) | 1.50 | (0.63-3.54) |
| >=35 | 3.10 | (1.55-6.18) | 3.86 | (1.38-10.8) | 2.74 | (1.17-6.42) |
| **Ethnicity** |  |  |  |  |  |  |
| White | 1 |  | 1 |  | 1 |  |
| Black African | 1.54 | (1.10-2.17) | 4.73 | (2.05-10.9) | 1.29 | (0.88-1.90) |
| Black Caribbean | 2.15 | (1.16-3.98) | 6.90 | (2.32-20.5) | 1.22 | (0.52-2.86) |
| Asian | 2.03 | (1.07-3.84) | 7.31 | (2.35-22.7) | 2.04 | (1.01-4.12) |
| Other | 0.57 | (0.20-1.63) | 4.31 | (1.06-17.5) | 0.54 | (0.19-1.55) |
| **Parity since diagnosis** |  |  |  |  |  |  |
| 1 | 1 |  | 1 |  | 1 |  |
| 2 | 0.63 | (0.48-0.83) | 0.89 | (0.59-1.34) | 0.73 | (0.52-1.03) |
| 3 | 0.59 | (0.43-0.81) | 0.99 | (0.64-1.54) | 0.54 | (0.36-0.83) |
| 4 or more | 0.49 | (0.33-0.73) | 0.85 | (0.50-1.45) | 0.67 | (0.42-1.08) |
| **Maternal risk factor for acquisition** | |  |  |  |  |  |
| Heterosexual | 1 |  | 1 |  | 1 |  |
| IDU | 1.62 | (0.56-4.68) | 3.81 | (0.78-18.6) | 1.02 | (0.23-4.47) |
| Other | 1.43 | (0.64-3.17) | 1.57 | (0.46-5.38) | 1.83 | (0.80-4.19) |
| Vertical | 1 |  | - |  |  |  |
| **CD4 in Pregnancy (cells/µL)** |  |  |  |  |  |  |
| ≥350 | 1 |  | 1 |  | 1 |  |
| <350 | 0.69 | (0.51-0.94) | 0.69 | (0.47-1.02) | 0.75 | (0.50-1.12) |
| **On Treatment at Conception** |  |  |  |  |  |  |
| No | 1 |  | 1 |  | 1 |  |
| Yes | 1.13 | (0.86-1.48) | 1.08 | (0.47-1.02) | 1.03 | (0.72-1.47) |
| **PI-based regimen in pregnancy** |  |  |  |  |  |  |
| No | 1 |  | 1 |  | 1 |  |
| Yes | 0.96 | (0.76-1.21) | 1.03 | (0.73-1.45) | 0.95 | (0.71-1.27) |
|  |  |  |  |  |  |  |
